# Characteristics of peripheral immune response induced by large-vessel occlusion in patients with acute ischemic stroke

**DOI:** 10.3389/fneur.2024.1512720

**Published:** 2024-12-10

**Authors:** Ling Ma, Bin Sun, Chenliu Fan, Juan Xiao, Maomao Geng, Jie Liu, Runze Jiang, Yang Jiang, Dianwei Liu

**Affiliations:** ^1^Department of Clinical Laboratory, The Second Hospital of Shandong University, Jinan, Shandong, China; ^2^Department of Stroke Center, Central Hospital Affiliated to Shandong First Medical University, Jinan, Shandong, China; ^3^Hematology Department, The Second Hospital of Shandong University, Jinan, Shandong, China; ^4^Department of Clinical Laboratory, Weihai Haida Hospital, Weihai, Shandong, China; ^5^Jinan Biomedical Industry Academy of Shandong First Medical University, Jinan, Shandong, China

**Keywords:** acute ischemic stroke, large vessel occlusion, T cell receptors repertoire, CD45, T cell

## Abstract

**Introduction:**

Despite improvements in the treatment of acute ischemic stroke (AIS), some patients still suffer from functional impairments, indicating the poor understanding of pathophysiologic process of AIS. Inflammation plays an important role in the pathophysiology of AIS. The purpose of the study was to investigate the peripheral inflammation in different subtypes of AIS.

**Methods:**

Here, retrospective data from AIS with large vessel occlusion (LVO) and small vessel occlusion (SVO), and healthy controls, were initially analyzed. Then, flow cytometry was performed to evaluate the levels of peripheral naïve and memory T-cells. Finally, we characterized the T cell receptors (TCR) repertoire using high-throughput sequencing.

**Results:**

Elevated levels of leukocytes, neutrophils, and neutrophil-to-lymphocyte ratio (NLR), and decreased levels of lymphocytes were found in LVO group than that in SVO group, which were correlated with the severity of LVO. In addition, higher percentages of both effector memory (Tem) and central memory (Tcm) T cells, and lower percentage of naïve T cells in CD4^+^ and CD8^+^ T cells, were found in LVO group than that in SVO and healthy groups. Moreover, impaired TCR diversity, and different abundances of V-J gene combinations and amino acid sequences, were found in LVO as compared with healthy group, which would be potential biomarkers for LVO diagnosis.

**Discussion:**

In conclusion, AIS with LVO can rapidly induce peripheral immune response, which provides new insight into the understanding of pathophysiology of AIS.

## Introduction

Acute ischemic stroke (AIS) is one of the leading causes of mortality and disability worldwide (1). It occurs due to brain ischemia resulting from the thrombosis of cerebral blood vessels (2), which can be mainly caused by large artery atherosclerosis (LAA) and small artery occlusion (SAO) according to the Trial of Org 10,172 in Acute Stroke Treatment (TOAST) classification (3). Although the outcomes of AIS have dramatically improved due to the effectiveness of endovascular therapy, these treatments are highly time-dependent and only a few patients with AIS could receive effective treatment in time (4). Most importantly, several strategies with regarding to AIS therapy have not been successfully translated into clinical application to date (5). These indicate that the pathological and physiological process contributing to neurological injury following AIS have not yet been fully understood.

Increasing evidence confirms that the activation of immune response is a crucial contributor to the pathophysiology of AIS (6, 7). Peripheral immune cells, such as neutrophils, lymphocytes, and monocytes, play important roles in the progression of AIS (8). In addition, the high neutrophil-to-lymphocyte ratio (NLR) is a potential predictor of poor functional outcome in patients with AIS (9). However, the changes of the peripheral components in different subtypes of AIS remains unclear.

The levels of lymphocytes were confirmed to be correlated with the outcome of AIS. Decreased number of lymphocytes was associated with worse pathological complete response rate of stroke (10), while increased proportion of lymphocytes had beneficial effects in AIS (11, 12). Among all the lymphocytes, T cells have been extensively studied because of their potency in both innate and adaptive immune responses (13). They are divided into CD4^+^ helper T cells, CD8^+^ toxic T cells, and regulatory T cells (Tregs) according to the different surface markers (14, 15), which play different regulatory roles in the pathophysiological process of AIS depending on their functional characteristics. The reduction of CD4^+^ or CD8^+^ T cells within 24 h after AIS leads to a decrease in the infarct size. In contrast, Tregs have protective effect on lowering infarct area and improving neurological function (16, 17). In addition, studies have shown that T cells could promote the deterioration of functional damage in the early stage but improve prognosis in the later stage of AIS, suggesting the different roles of T cell subsets in AIS (18, 19). Moreover, immune cell infiltration analysis suggested that T cell subsets with relevant genes can be identified as the diagnostic biomarkers in AIS (20). Therefore, it is essential to investigate the functions of different T cell subsets in AIS, which will provide new insights into the pathophysiological mechanisms of AIS. Recent years, a new group of T cells with CD45 surface markers has been discovered, which can be divided into two new subgroups: CD45RA^+^ naïve T cells and CD45RO^+^ memory T cells (21). Previous studies have confirmed the involvement of CD45 subsets in different diseases, such as sepsis and T-cell lymphoma (21, 22). However, it is still unknown whether CD45RA^+^ and CD45RO^+^ T cells are involved in the progression of AIS.

T cells initiate their major functions through T cell receptors (TCRs), which are produced by somatic DNA recombination of multiple gene segments (23). The diversity is generated by the random rearrangement of the variable (V), diversity (D), and joining (J) segments of TCR genes, which are central components of the adaptive immune system. TCR sequences are individual and have complex genetics due to VDJ recombination (24). Analysis of the TCR repertoire can provide a better understand of immune-mediated responses to infections, malignancies, and immunological disorders, including neuroinflammatory diseases. Based on technological advances in high-throughput sequencing (HTS), millions of TCR sequences can be used to assess clonal expansion and diversity in the peripheral blood of the multiple sclerosis (MS) patients (25, 26). The unique sequences will be valuable biomarkers for immune-mediated disease diagnosis, prognosis, and treatment response. Although a few studies have focused on TCR or characteristics of TCR repertoires in brain or peripheral blood of AIS (27–29), these studies did not distinguish the changes of TCR characteristic in the subtypes of AIS.

In the present study, we initially analyzed the circulating data retrospectively in patients with AIS, which were divided into large-vessel occlusion (LVO) and small-vessel occlusion (SVO) by imaging methods. Then, peripheral blood samples of patients with LVO and SVO were collected to detect proportional changes of CD45RA^+^ and CD45RO^+^ T cells. Finally, the TCR repertoire was analyzed to identify the unique immune response in AIS with LVO.

## Materials and methods

### Research ethics

The study was conducted according to the guidelines of the Declaration of Helsinki, and approved by the Ethics Committee of Jinan Central Hospital Affiliated to Shandong First Medical University (No. SZR2021-006-01) and The Second Hospital of Shandong University (No. KYLL-2021 (KJ)P-0300).

### Study population

Retrospective data from 368 patients with AIS (≥18 years old), recruited from both Central Hospital Affiliated to Shandong First Medical University (*n* = 312) and The Second Hospital of Shandong University (*n* = 56) between September 2022 to December 2023, were analyzed for peripheral clinical characteristics. The AIS patients were divided into large vessel occlusion (LVO, *n* = 161) and small vessel occlusion (SVO, *n* = 207) using magnetic resonance imaging (MRI), computed tomography angiography (CTA), brain magnetic resonance angiography (MRA), and/or digital subtraction angiography (DSA) (30). Exclusion criteria: (1) only received MRI without further brain imaging; (2) had severe other disease, such as liver or kidney dysfunction, cardiac impairment; (3) had severe inflammatory conditions. The age- and sex-matched healthy participations (*n* = 167), which were confirmed to have no cerebrovascular disease or other sever conditions in the physical Examination Department of The Second Hospital of Shandong University over the same period, were included as the control group.

### Clinical data collection

Venous blood samples were collected within the first 24 h of stroke onset. Blood glucose, low-density lipoprotein (LDL) cholesterol, high-density lipoprotein (HDL) cholesterol, triglyceride (TG), and counts of leukocyte, neutrophile, and lymphocyte as well as the proportion of neutrophile and lymphocyte, were analyzed. The NLR was calculated as the ratio of the absolute neutrophile counts to the absolute lymphocyte counts. The stroke severity at onset was evaluated using National Institutes of Health Stroke Scale (NIHSS) (31).

### Flow cytometry analysis

To determine the phenotype of T cells, 18 patients with LVO and 17 patients with SVO, aged from 32 to 82 years, were recruited from both Central Hospital Affiliated to Shandong First Medical University (LVO, *n* = 8) and The Second Hospital of Shandong University (SVO, *n* = 17; LVO, *n* = 10) from May to December 2023. Peripheral anticoagulant blood samples from patients were obtained within 24 h of AIS onset at the Department of Clinical Laboratory. The samples of 22 healthy controls were collected at the same time. The whole blood of each sample was mixed gently and transferred into five groups (100 μL/tube): (1) labeled with APC-conjugated mouse anti-human CD3 (#317318, Biolegend, San Diego, CA, United States) and FITC-conjugated mouse anti-human CD56 (#304604, Biolegend) antibody; (2) labeled with APC-conjugated mouse anti-human CD3, FITC-conjugated mouse anti-human CD4 (#300506, Biolegend), and PE-conjugated mouse anti-human CD8 (#344706, Biolegend) antibody; (3) labeled with PE-conjugated mouse anti-human CD4 (#300508, Biolegend), PerCP-conjugated mouse anti-human CD45RA (#304156, Biolegend), FITC-conjugated mouse anti-human CD45RO (#304204, Biolegend), and APC-conjugated mouse anti-human CCR7 (#353214, Biolegend) antibody; (4) labeled with PE-conjugated mouse anti-human CD8, PerCP-conjugated mouse anti-human CD45RA, FITC-conjugated mouse anti-human CD45RO, and APC-conjugated mouse anti-human CCR7 antibody; (5) the isotype control tube labeled with APC/FITC/PE/PerCP rat anti-human IgG antibody (#410712, #410720, #410707, #410710, Biolegend). Five microliter of each antibody was added into the corresponding tube and incubated for 20 min. Then, 1 mL erythrocyte lysing buffer (#555899, BD Biosciences, San Jose, CA, United States) was added and incubated at 37°C for 5 min. After centrifugation for 5 min, the cells were resuspended and washed with 1 mL phosphate buffer solution (PBS). Finally, after being suspended with 0.5 mL PBS, the cells were detected by flow cytometry (FACS Aria III; BD Biosciences). The gating strategy applied for the enumeration of T cells is shown in Supplementary Figure S1. Peripheral whole blood cells, including neutrophils, lymphocytes, monocytes, and red blood cells, can be divided into different populations based on cell size and granularity, as measured by forward scatter (FSC) and side scatter (SSC) characteristics, respectively. Lymphocyte were gated on the basis of FSC and SSC characteristics for the following research. The cells were analyzed by using FlowJo VX10 software (TreeStar, Ashland, OR, United States).

### HTS of TCR repertoire

Peripheral blood samples were collected into EDTA vacutainer tubes at volumes more than 2 mL. Peripheral blood mononuclear cells (PBMCs) were isolated from whole blood samples using Ficoll density-gradient separation lysis (LTS1077-1, TBD, Tianjin, China) according to the instructor. Total RNA was extracted from PBMCs using RNAsimple Total RNA Kit (#DP419, Tiangen Biotech, Beijing, China). RNA concentration was evaluated using a NanoDrop ND-2000 spectrophotometer (Thermo Scientific, United Kingdom). cDNA synthesis and multiplex PCR amplification of the complementary-determining region 3 (CDR3) in the TCR *β*-chain were performed together using the Immune Repertoire Library Preparation Kit (Geneway, Jinan, China) following a protocol described in a previous study (32). TCR libraries were sequenced on DNBSEQ-T7 platform (MGI, Shenzhen, China), generating paired-end short reads with 150 bp in length.

### Sequencing data preprocessing

The sequencing data were stored in FASTQ format, in which raw reads were demultiplexed according to the sequences of index primers corresponding to different samples. The low-quality sequences were discarded for quality control. The remainders were mapped into V, D, and J gene segments of TCR *β*-chain using the MiXCR software (version 3.0.6) with default parameters for sequencing alignment and clonotype assembly (33). TCR reference gene data were downloaded from the IMGT database^1^. The frequency of each TCR β-clonotype was further converted into rpm (reads per million) for standardization. The diversity of samples was evaluated based on D50 Diversity index and UT index. The diversity from the cumulative 50% of the total CDR3 detected in the sample was measured using the D50 index (34). The UT index was ranged from 0 to 1, and it was calculated based on the previous study (35).

### Statistical analyses

Data were analyzed using GraphPad Prism software (Version VIII, La Jolla, CA, United States) or R software (version 4.0.2). Continuous data were presented as means ± standard deviation (SD). In contrast, categorical variables were presented as numbers and percentages. In the analysis of retrospective data and flow cytometry, the one-way analysis of variance (ANOVA) or Kruskal–Wallis test was used for comparisons between more than two groups based on data distribution and homogeneity. One-way ANOVA followed by Tukey test was used when the data showed normal distribution and variance homogeneity, otherwise Kruskal–Wallis test was applied. For continuous variables in the TCR repertoire, Student’s *t-*test was used for comparison between two groups, and the correlation between NIHSS and levels of peripheral blood cells, or between NIHSS and TCR clonotypes expression, was assessed using Pearson’s test. The Chi-Square test or Fisher’s exact test was used to analyze categorical variables. *p* < 0.05 was considered the threshold for statistical significance.

## Results

### Participation clinical characteristics

To determine the peripheral immune responses in different subtypes of AIS, we initially analyzed the retrospective data from patients with LVO, SVO, and healthy controls. The baseline demographic and clinical characteristics are shown in Table 1 and Supplementary File 1. No significant differences were found among the three groups in baseline characteristics including age and gender. As risk factors of AIS, lower levels of HDL cholesterol, and high levels of both blood glucose and triglyceride were found in patients with LVO and SVO groups than in healthy controls (*p* < 0.0001). No significant change was found in levels of low-density lipoprotein (LDL) cholesterol after AIS.

**Table 1:** Baseline and clinical characteristics of patients with AIS and healthy controls.

Valuables	LVO (*n* = 161)	SVO (*n* = 207)	Healthy (*n* = 167)	*p*-value
Gender, male, *n* (%)	103 (63.98)	125 (60.39)	102 (61.08)	0.7668
Age, years (mean ± SD)	66.09 ± 12.29	65.11 ± 10.4	63.87 ± 7.986	0.1496
Glucose, mmol/L (mean ± SD)	7.527 ± 3.314	7.31 ± 2.703	5.175 ± 0.6554	<0.0001
HDL, mmol/L (mean ± SD)	1.105 ± 0.2735	1.108 ± 0.4093	1.407 ± 0.2397	<0.0001
LDL, mmol/L (mean ± SD)	2.429 ± 0.8143	2.67 ± 0.7394	2.596 ± 0.5713	0.0512
Triglycerides, mmol/L (mean ± SD)	1.295 ± 0.7852	1.573 ± 1.204	0.964 ± 0.4057	<0.0001

HDL, high density lipoprotein; LDL, low density lipoprotein.

**Figure 1:**
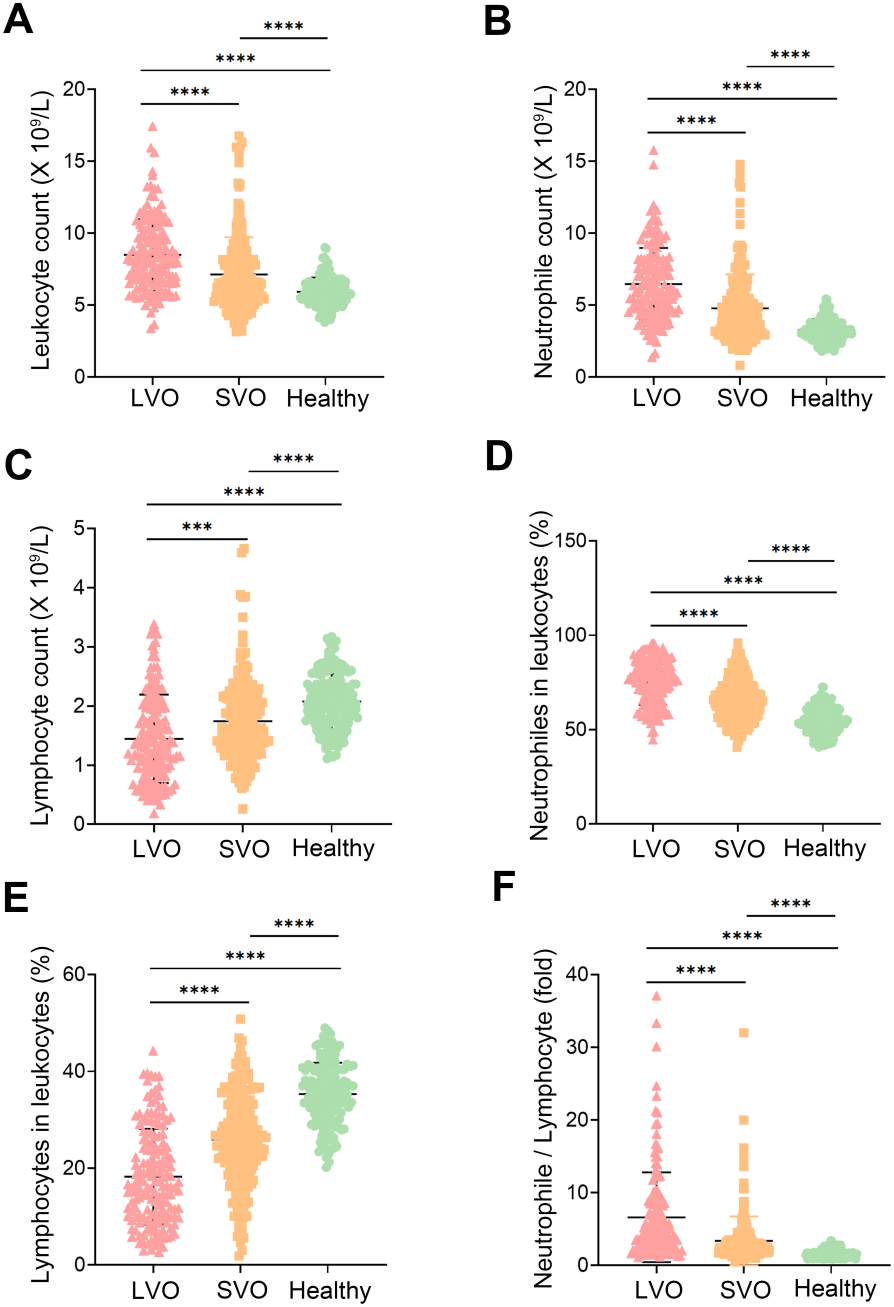
Participation characteristics. Leukocyte **(A)**, neutrophile **(B)**, and lymphocyte **(C)** counts in the peripheral blood of AIS with LVO and SVO, and healthy controls. The ratio of neutrophiles **(D)** and lymphocytes **(E)** to leukocytes in the peripheral blood of AIS with LVO and SVO, and healthy controls. **(F)** The ratio of neutrophile to lymphocyte in the peripheral blood of AIS with LVO and SVO, and healthy controls. (***p < 0.001, ****p < 0.0001).

The laboratory parameters in patients with LVO and SVO were significantly different from those of healthy controls (Figure 1 and Supplementary File 1). The leukocyte count in the patients’ groups was higher than that in the healthy control group (Figure 1A, *p* < 0.0001), which was mainly due to an elevated neutrophile count (Figure 1B, *p* < 0.0001). In contrast, the lymphocyte count decreased in patients with AIS compared to the healthy control group (Figure 1C, *p* < 0.0001). An increased ratio of neutrophile to leukocyte and a decreased ratio of lymphocyte to leukocyte were also observed in patients with AIS (Figures 1D,E, *p* < 0.0001). As NLR has been reported to be a useful marker of inflammation, we also compared NLR in the three groups. Consistent with the previous study, NLR was higher in patients than that in healthy controls (Figure 1F, *p* < 0.0001). Interestingly, we found higher counts of leukocyte (Figure 1A, *p* < 0.0001) and neutrophile (Figure 1B, *p* < 0.0001), and elevated ratio of neutrophile to leukocyte (Figure 1D, *p* < 0.0001) and NLR (Figure 1F, *p* < 0.0001) in LVO group than that in SVO group. In contrast, lymphocyte count (Figure 1C, *p* = 0.0003) and ratio of lymphocyte to leukocyte (Figure 1E, *p* < 0.0001) in LVO group were decreased as compared with that in SVO group. These results suggest that AIS with LVO can rapidly induce more severe immune response in the peripheral blood.

### Correlation between peripheral blood cells and NIHSS

**Figure 2:**
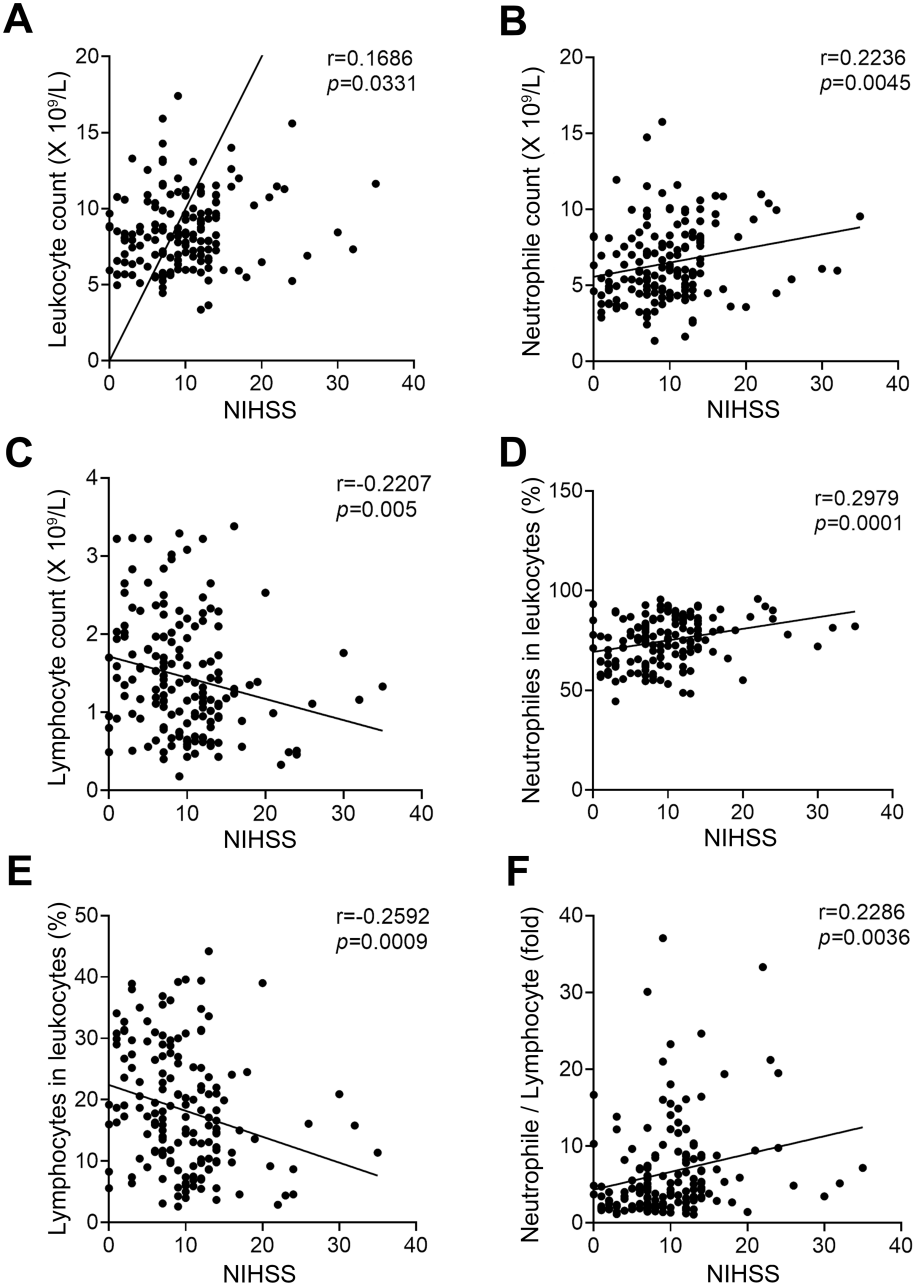
Correlation between NIHSS and circulating blood cells in patients of AIS with LVO. The correlation between NIHSS and leukocyte **(A)**, neutrophile **(B)**, and lymphocyte **(C)** counts. The correlation between NIHSS and the ratio of neutrophiles **(D)** and lymphocytes **(E)** to leukocytes. **(F)** The correlation between NIHSS and the ratio of neutrophile to lymphocyte.

To determine whether there was a relationship between the expression levels of peripheral blood cells and the severity of AIS, we analyzed the correlation between the peripheral laboratory data and NIHSS in LVO group and SVO group, respectively. As shown in Figure 2 and Supplementary File 1, the counts of leukocytes (Figure 2A, *r* = 0.1686, *p* = 0.0331) and neutrophiles (Figure 2B, *r* = 0.2236, *p* = 0.0045), and the percentage of neutrophiles (Figure 2D, *r* = 0.2979, *p* = 0.0001) as well as NLR (Figure 2F, *r* = 0.2286, *p* = 0.0036) were positively correlated with NIHSS in the LVO group. In contrast, both the count and percentage of lymphocytes were inversely correlated with NIHSS in the LVO group (Figure 2C, *r* = −0.2207, *p* = 0.005; Figure 2E, *r* = −0.2592, *p* = 0.0009). However, no relationship was found between the levels of peripheral blood cells and NIHSS in the SVO group (data not shown). These results suggest that the expression levels of peripheral immune cells can more specifically reflect the severity of patients with AIS caused by LVO.

### Phenotype analysis of peripheral T cells

**Figure 3:**
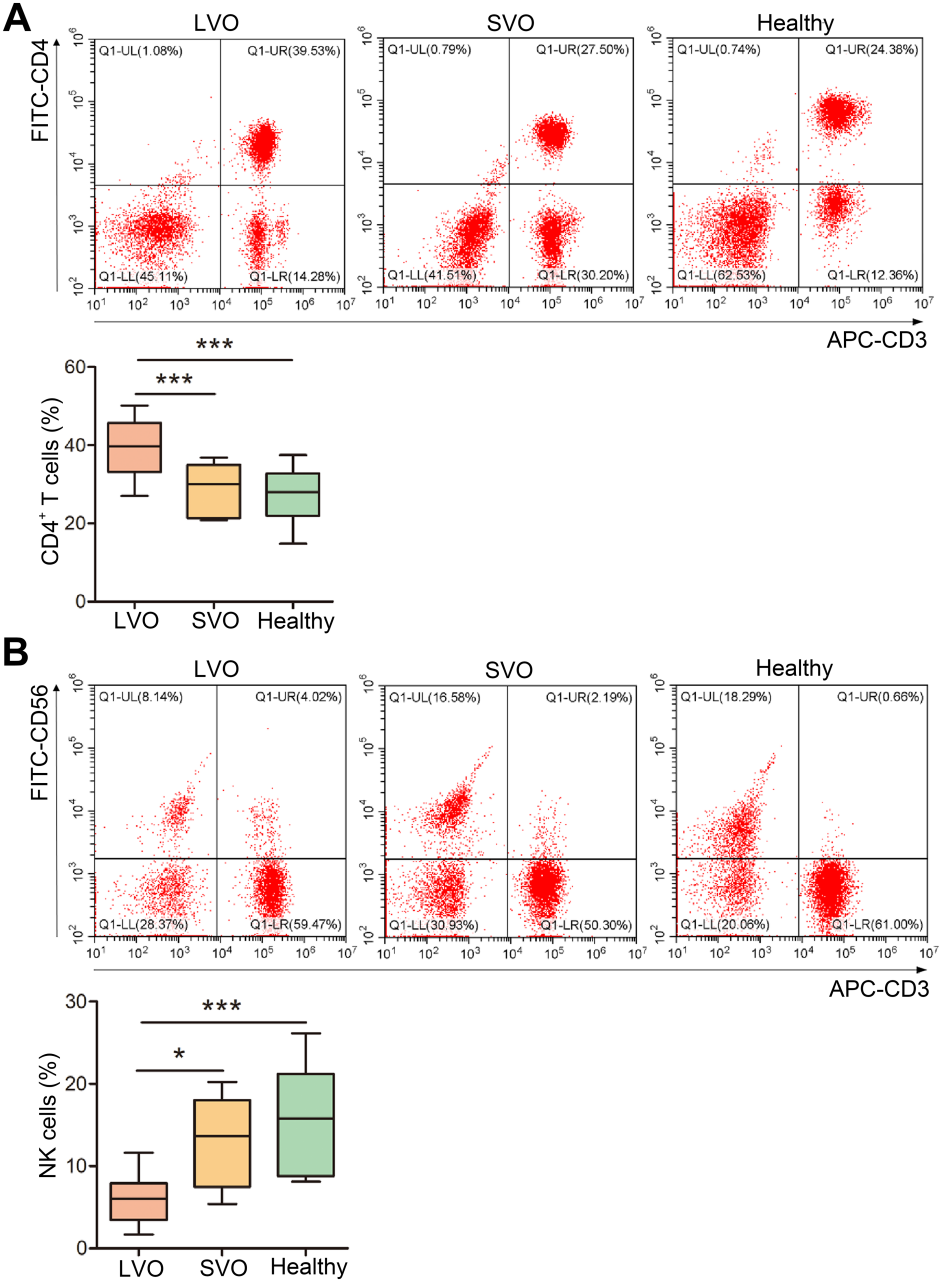
Changes in T-cell subtypes. **(A)** The percentage of CD4^+^ T cells was determined by flow cytometry. **(B)** The percentage of NK cells was determined using flow cytometry (*p < 0.05, ***p < 0.001).

T cells play essential roles in immune response. To detect the peripheral phenotype of T cells in AIS with different subtypes, flow cytometry analysis was performed. As shown in Figure 3 and Supplementary File 2, although the total number of peripheral lymphocytes was significantly reduced in patients with AIS, the proportion of CD4^+^ T cells was higher in the LVO group than in the SVO and healthy control groups (Figure 3A, *p* < 0.001). In contrast, the proportion of NK cells decreased in the LVO group as compared with the SVO group (Figure 3B, *p* < 0.05) and control group (Figure 3B, *p* < 0.001). No significant difference was found between the SVO group and control group. These results indicate that AIS with LVO can rapidly enhance the adaptive immune response mediated by T cells.

**Figure 4:**
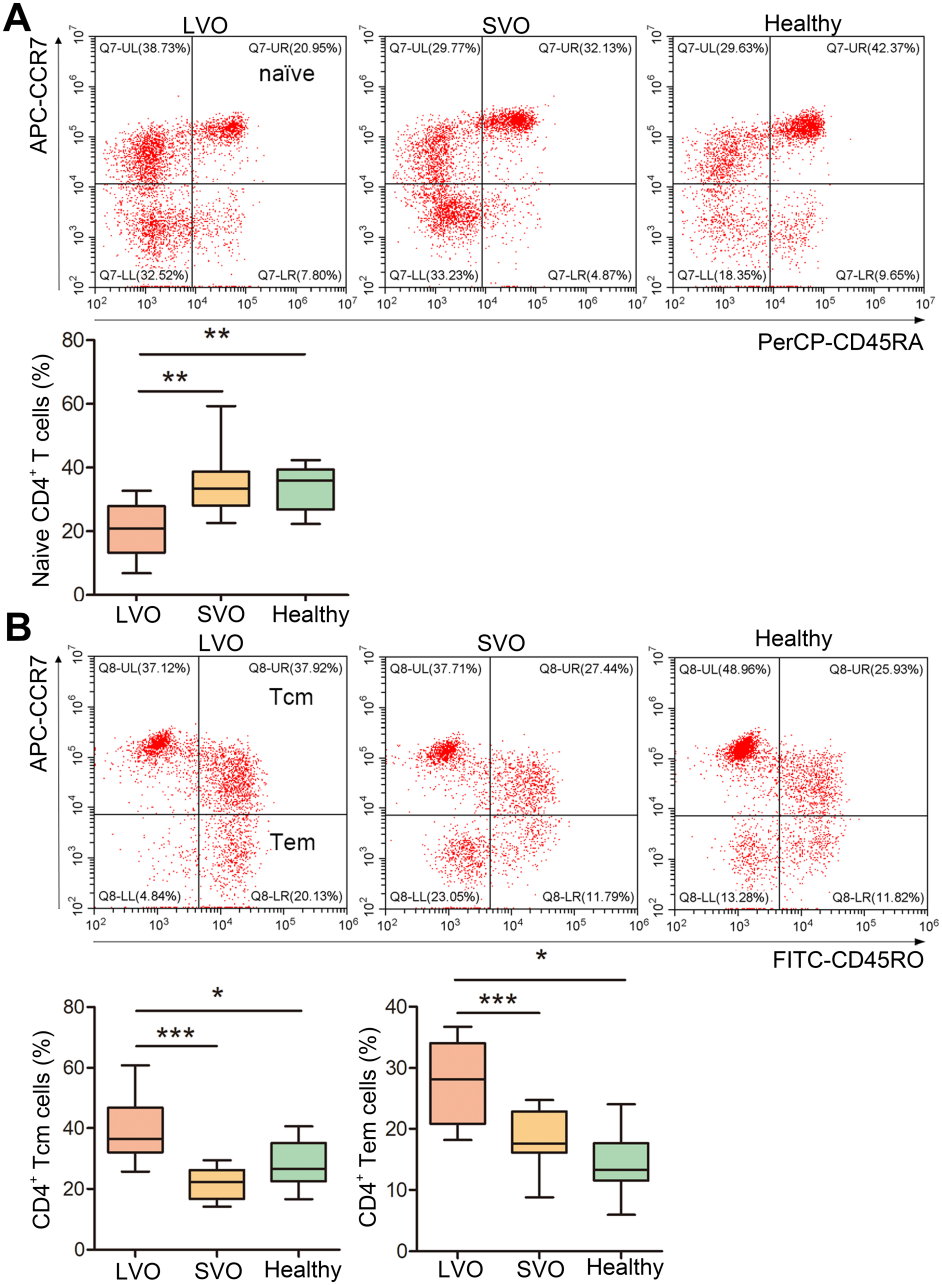
Changes in naïve, Tcm, and Tem CD4^+^ T cells. **(A)** The percentage of naïve CD4^+^ T cells was determined by detecting CD45RA^+^CCR7^+^ cells among the CD4^+^ T cells. **(B)** The percentage of Tcm CD4^+^ T cells was investigated by detecting CD45RO^+^CCR7^+^ cells among the CD4^+^ T cells. The percentage of Tem CD4^+^ T cells was investigated by detecting CD45RO^+^CCR7^−^ cells in CD4^+^ T cells. (*p < 0.05, **p < 0.01, ***p < 0.001).

**Figure 5:**
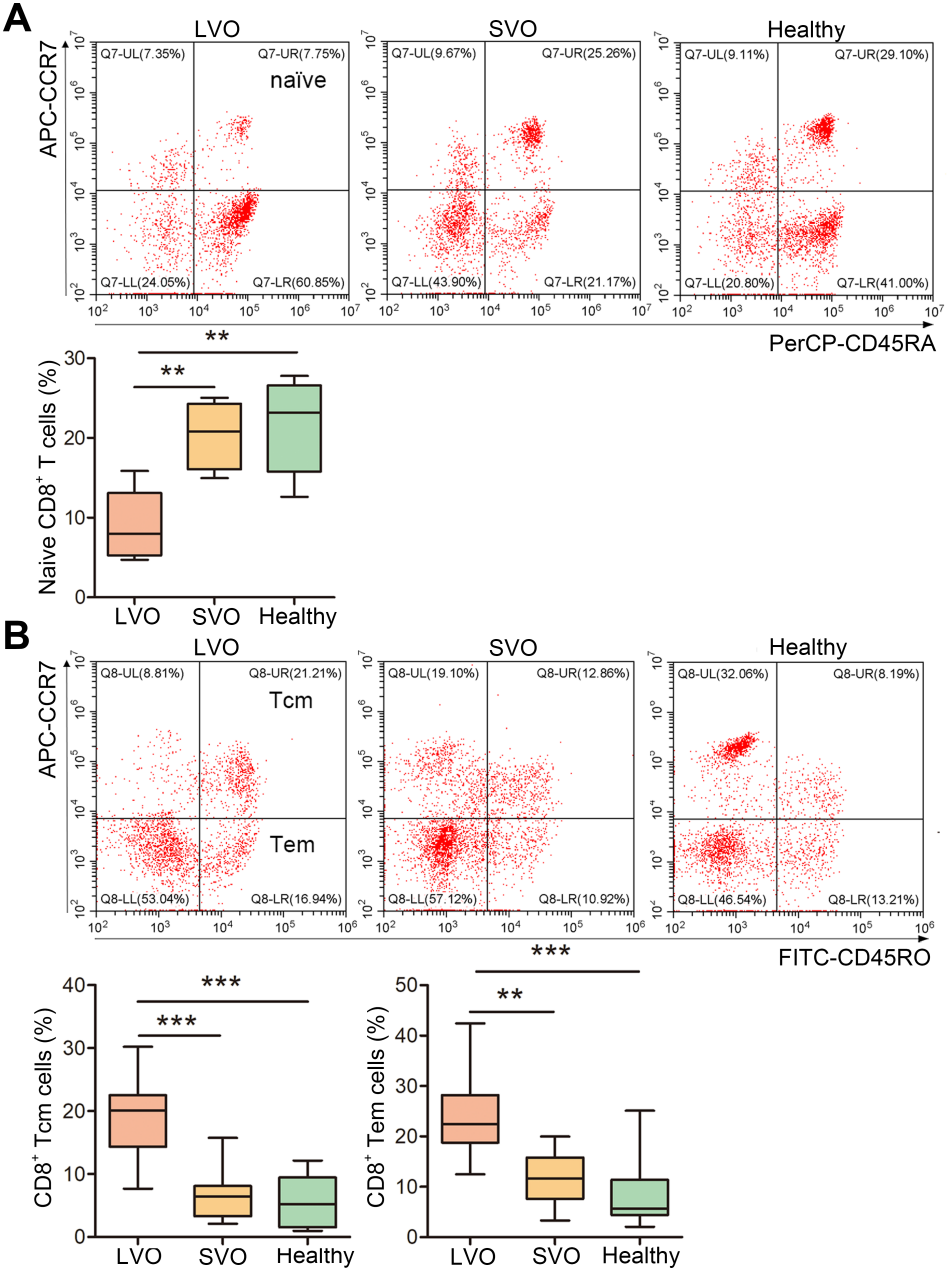
Changes in naïve, Tcm, and Tem levels in CD8^+^ T cells. **(A)** The percentage of naïve CD8^+^ T cells was investigated by detecting CD45RA^+^CCR7^+^ cells among CD8^+^ T cells. **(B)** The percentage of Tcm CD8^+^ T cells was investigated by detecting CD45RO^+^CCR7^+^ cells among CD8^+^ T cells. The percentage of Tem CD8^+^ T cells was investigated by detecting CD45RO^+^CCR7^−^ cells in CD8^+^ T cells. (**p < 0.01, ***p < 0.001).

To further analyze the immune response after AIS, naïve, effector memory T (Tem), and central memory T (Tcm) of CD4^+^ and CD8^+^ T cells were, respectively, detected in LVO and SVO subtypes. As shown in Figure 4 and Supplementary File 2, the percentage of CD45RA^+^CCR7^+^ in CD4^+^ (naïve CD4^+^) T cells decreased (Figure 4A, *p* < 0.01), while the percentage of CD45RO^+^CCR7^+^ (Tcm, Figure 4B, *p* < 0.001) and CD45RO^+^CCR7^−^ (Tem, Figure 4B, *p* < 0.001) in CD4^+^ T cells increased in LVO group as compared with SVO and control groups, suggesting a decrease in naïve CD4^+^ T cells and an increase in Tcm and Tem CD4^+^ T cells after AIS with LVO. Although no significant difference was found in the percentage of total CD8^+^ T cells, similar changes were found in CD45RA^+^CCR7^+^ (Figure 5A, *p* < 0.01, Supplementary File 2), CD45RO^+^CCR7^+^ (Figure 5B, *p* < 0.001), and CD45RO^+^CCR7^−^ (Figure 5B, *p* < 0.01) in CD8^+^ T cells, as in CD4^+^ T cells of patients with LVO. No significant difference was found between the SVO and healthy control groups in naive, Tcm, and Tem cells. These results suggest that only AIS with LVO can stimulate the transformation of T cells into memory T cells.

### The characteristics of TCR repertoires in AIS patients with LVO

**Figure 6:**
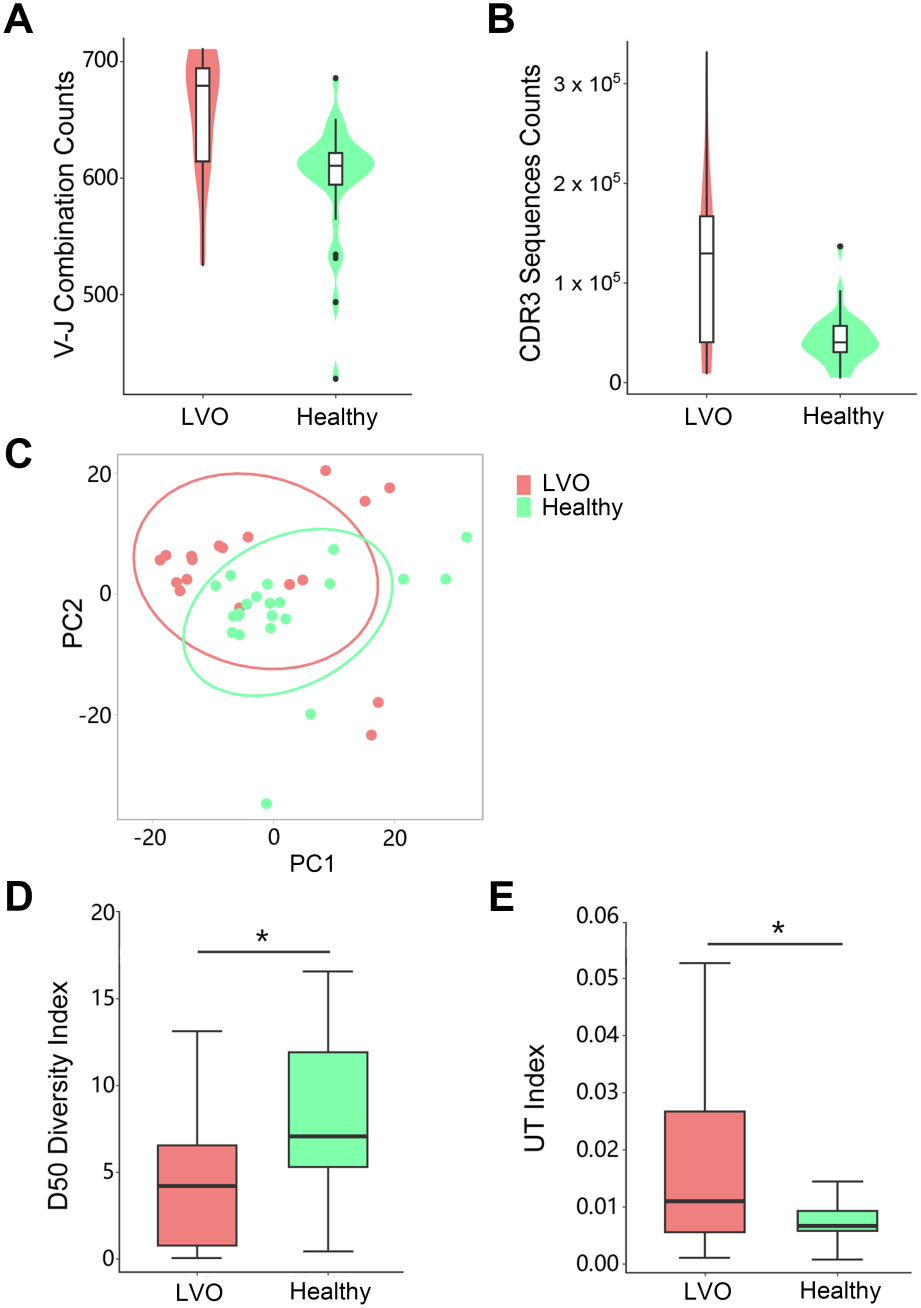
Quantity and diversity of the TCR repertoire: the number of unique V-J combinations **(A)** and counts of unique CDR3 sequences **(B)** were determined. **(C)** Principal component analysis of the AIS with LVO (red) and healthy groups (green). *X*-axis and *Y*-axis represent principal component 1 (PC1) and principal component 2 (PC2), respectively. The D50 Diversity Index **(D)** and UT Index **(E)** show the diversity of the TCR repertoire in the AIS with LVO and healthy groups. The violin chart and box plot show the data distribution with the minimum, first quartile, median, third quartile, and maximum (*p < 0.05).

Given the changes of T cells above, we sought to further determine the T cells’ characteristics of LVO. As no changes were found in T cells between SVO group and healthy control group, PBMCs were isolated from peripheral blood and TCR repertoire sequencing analysis were performed in AIS patients with LVO and healthy controls. We assessed TCR sequences and identified V-J combinations at the transcription level. The results showed that the number of V-J combinations (Figure 6A, *p* = 0.0028) and the TCR sequences in CDR3 (Figure 6B, *p* = 0.0018, Supplementary File 3) were more abundant in AIS with LVO group than in control group. Notably, consistent with the increased memory T cells, these results indicate a dramatic increase in immunological activity in the number of immunological items during the pathological process of AIS with LVO.

In addition, we performed a Principal Component Analysis (PCA) on the V-J combination frequency profile. As shown in the Figure 6C and Supplementary File 3, there was a significant difference between AIS patients with LVO and healthy controls in the sample cluster. In summary, we constructed a phenotype of LVO with immunological tendencies compared to healthy controls. TCR expression profiles were subsequently analyzed to assess the systemic immune responses mediated by T cells.

Next, we estimated the diversity of TCR clonotypes in each sample by calculating the D50 Diversity and UT index, irrelevant to the variation of sample sequencing depth. A lower D50 Diversity index was observed in AIS patients with LVO than in healthy controls (Figure 6D, *p* = 0.0498). In contrast, the UT index was higher in AIS patients with LVO than in healthy controls (Figure 6E, *p* = 0.02, Supplementary File 3), indicating that AIS with LVO could decrease the diversity of TCR profiles as compared with healthy controls.

### Diversity of TCR repertoires and usage frequency of V-J gene combinations in AIS patients with LVO

**Figure 7:**
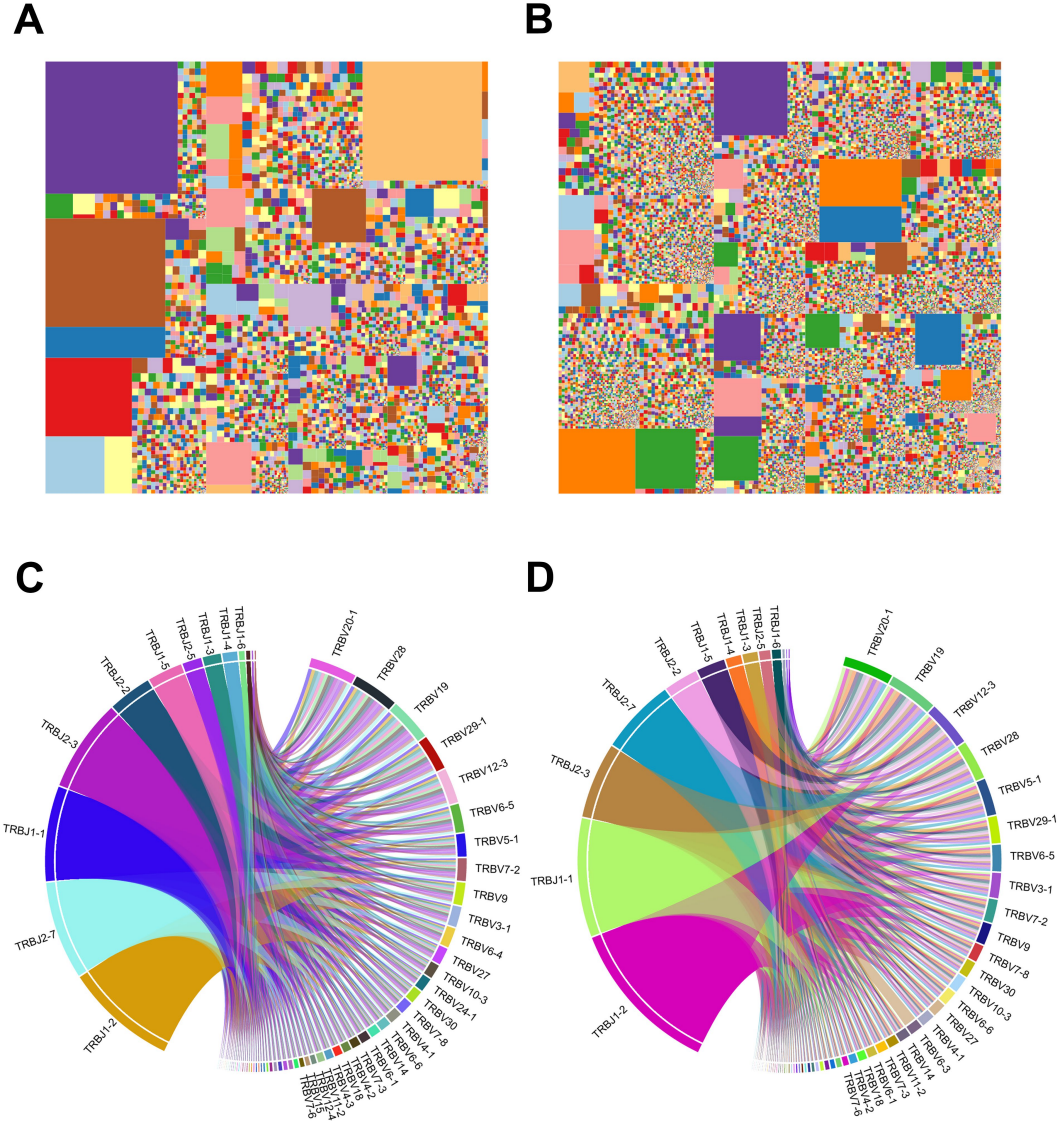
Samples’ immunological characteristics. **(A,B)** TCR sequence abundance and usage frequency of V-J combinations in patients with LVO. Tree-map of module CDR3 sequence abundance in samples from the AIS with LVO group **(A)** and healthy group **(B)**. **(C,D)** Circos plots of the V-J gene combination usage frequency in samples from the AIS with LVO group **(C)** and healthy group **(D)**. The left half-circle indicates the J gene and the right half-circle indicates the V gene. The length of sectors represents the relative usage frequency of the V genes or the J genes.

We further performed a characteristic analysis to reveal the specificity of TCR sequence abundance in AIS patients with LVO and healthy controls. As shown in Figures 7A,B, the whole tree-map represented the average immune status of samples based on the abundance of CDR3 sequences. Each chip represented one CDR3 sequence’s abundance. The larger of the color chip, the higher abundance of this sequence. Meanwhile, large color chips led to a decrease in the quantity of chips, which indicated the reduction of diversity. We found more large-colored chips in AIS patients with LVO (Figure 7A) than in controls (Figure 7B and Supplementary File 4), which also illustrated the high-abundance sequences and poor TCR diversity in LVO patients.

As the most variable components of TCR sequence, V and J segments play a crucial role in targeting a wide range of pathogenic process and the combination of V-J segments are the primary focus of many TCR-related studies. The comparison of V-J segments could reveal their contributions to the progression of AIS, and help to explain the differences in immune status between different groups. Furthermore, Circos plots was used to show the usage of V-J gene combinations. In the Circos plots, the length of the sectors represents the relative usage frequency of the V or J genes, while the width of the links connecting the V and J genes represents the relative usage frequency of the V-J combinations. Nevertheless, patients with LVO (Figure 7C) showed the similar average frequency of the use of V-J gene combinations as healthy controls (Figure 7D and Supplementary File 4). These results further indicated that the TCR diversities in AIS patients with LVO were induced by the high abundance of VDR3 sequences.

### Different abundances of V-J gene combinations in AIS patients with LVO

**Figure 8:**
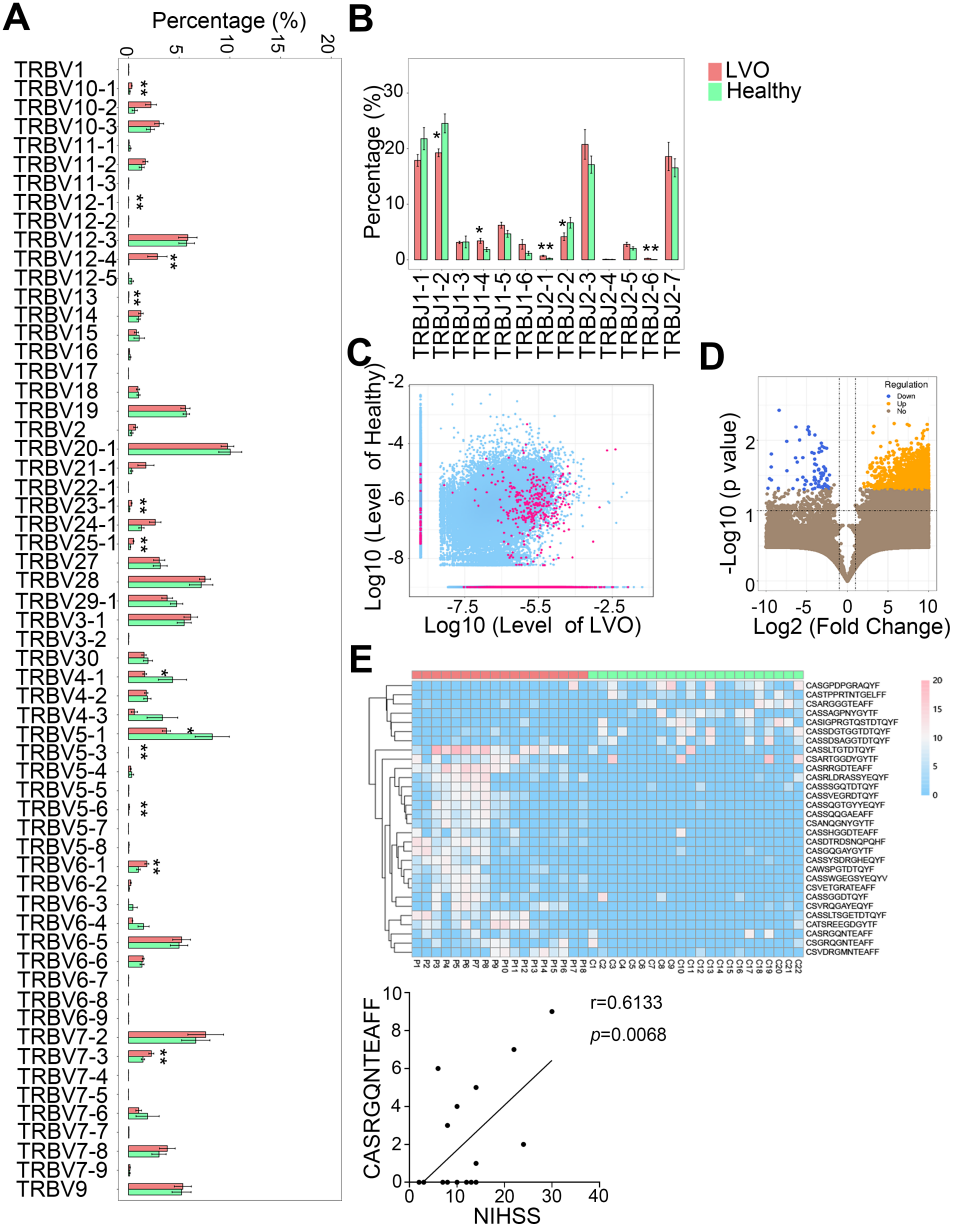
Differential abundances of the V and J gene segments and CDR3 sequences between the AIS with LVO and healthy groups. The relative abundance of V gene **(A)** and J gene **(B)** in the two groups. **(C)** Scatter plot showing differential abundance of CDR3 sequences in LVO and healthy groups (red, different CDR3 sequences; blue, CDR3 sequences with no significant difference). The *X*- and *Y*-axis represents the log-transformed mean of the relative abundance of the healthy and LVO group, respectively. **(D)** The volcano map shows the different clones between the LVO and healthy groups (yellow with increased abundance and blue with decreased abundance). The *X*- and *Y*-axes represent the log transformed *p*-value and fold changes, respectively. **(E)** Thirty differentially expressed amino acid clonotypes are shown as a heatmap. The *X*- and *Y*-axis represent samples and expression levels of CDR3 sequences, respectively. **(F)** The correlation between NIHSS and the unique amino acid clonotype (*p < 0.05, **p < 0.01).

We then determined the different abundance of V-J gene combinations in AIS patients with LVO from healthy controls. A total of 63V and 14J gene segments were identified in all samples. Compared with the control group, a significantly lower percentage of TRBV4-1 and TRBV5-1, and a higher percentage of TRBV5-3, TRBV5-6, TRBV6-1, TRBV7-3, TRBV10-1, TRBV12-1, TRBV12-4, TRBV13, TRBV23-1, and TRBV25-1 were found in the AIS with LVO group (Figure 8A, *p* < 0.05, *p* < 0.01). Moreover, a significantly lower percentage of TRBJ1-2 and TRBJ2-2, but a higher percentage of TRBJ1-4, TRBJ2-1, and TRBJ2-6 were found in the AIS with LVO group than the healthy group (Figure 8B, *p* < 0.05, *p* < 0.01, Supplementary File 5). These results indicated that LVO induced different abundance of V-J gene combinations.

We further analyzed the abundance of CDR3 sequences between AIS with LVO and healthy groups. There were 734 upregulated and 49 downregulated amino acid clonotypes between the AIS with LVO and healthy group (Figures 8C,D). In addition, 30 differentially expressed amino acid clonotypes, were found in at least 10 samples (Figure 8E). Among these clonotypes, the expression levels of one amino acid clonotypes (CASRGQNTEAFF) was found to be positively correlated with NIHSS (Figure 8F, *r* = 0.6133, *p* = 0.0068), suggesting that the expression level of this amino acid clonotypes was related to the severity of AIS with LVO.

### The prediction model for AIS with LVO

**Figure 9:**
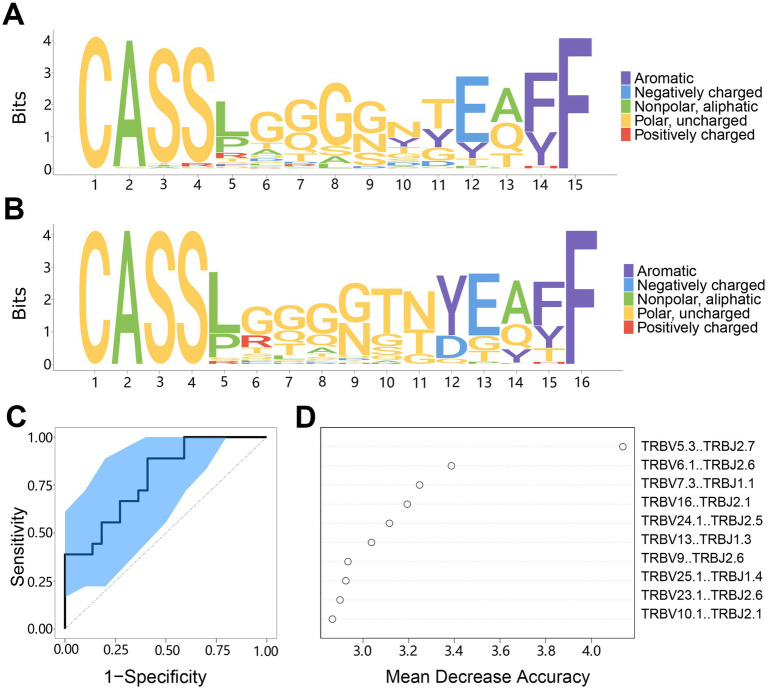
The motif specificity of TCR repertoires and prediction model system for AIS with LVO. **(A)** Motif diagram of the CDR3 sequences in AIS with LVO group. **(B)** Motif diagram of the CDR3 sequences in the healthy group. **(C)** ROC curve showing the classification effect of the LVO prediction model. The ordinate is the true-positive rate (sensitivity), and the abscissa is the false-positive rate (1-specificity). **(D)** The top 10 segments of V-J combinations that influenced the model effect. The mean decrease in accuracy is a rating index, and its value positively correlates with the effect on the model.

As the different abundance of TCR sequences between the AIS with LVO group and healthy group, we next want to build a diagnostic model to predict AIS with LVO. We firstly aligned the top 50 abundant CDR3 sequences with the indicated length to create a motif diagram. The results showed a significant difference in the motifs between the AIS with LVO group (Figure 9A) and the healthy group (Figure 9B). This suggests that selecting a suitable amino acid sequence can distinguish AIS patients with LVO from healthy controls.

Then, we created a model using the random forest method to predict AIS with LVO based on differences in TCR repertoire characteristics. We changed the settings from 0.2 to 0.3 to improve the accuracy, while lowering the fault tolerance rate to stabilize the classification function of the model. We then evaluated the model classification effect in predicting AIS patients with LVO. The results showed that the distribution of the ROC curve was relatively smooth, and the leave-one-out cross-validation produced an area under the curve (AUC, 95%CI: 0.519–0.981, Figure 9C). Additionally, we evaluated the V-J combinations that affected the model assessment effect and discovered 10 combinations that made the largest contributions to the model (Figure 9D). These results indicate that the model can distinguish between patients with LVO and healthy controls, which provides the possibility of developing TCR biomarkers for the early diagnosis of AIS with LVO.

## Discussion

Although endovascular therapy is effective for AIS, some patients still suffer from permanent disability. From the retrospective data, we firstly found that the changes of peripheral blood cells were correlated with the severity of AIS with LVO but not SVO. Using flow cytometry, we found that AIS with LVO enhanced the peripheral adaptive immune response by increasing the percentage of Tcm and Tem cells. Furthermore, TCR repertoire sequencing analysis showed that TCR diversity was impaired in patients with LVO, although the number of V-J combinations and CDR3 sequences increased. Together with the flow cytometry results, these findings suggest that AIS with LVO could induce an adaptive immune response, accompanied by a lack of comprehensive immunological activity, owing to the specific immune response to disease. Importantly, we found different abundances of V-J gene combinations and amino acid clonotypes between the AIS with LVO and control groups, which could be used as diagnostic biomarkers for AIS. This study will provide new insights into the pathophysiological process of AIS.

Human and animal studies have confirmed that AIS can lead to immediate activation of local immune cells and prompt mobilization of peripheral immune cells in the first hours and up to days after stroke (8, 36). Although studies have confirmed that the peripheral neutrophiles and NLR are closely related to the prognosis of AIS, few studies have focused on their roles on different subtypes of AIS. Our results confirmed that the changes of peripheral immune cells were more obvious in the LVO group than that in SVO group. In addition, these changes have a correlation with NIHSS in the LVO but not SVO group, suggesting that the peripheral immune changes can more specifically related to the severity of AIS patients with LVO. To our knowledge, this is the first study showing the relationship between the peripheral components and different AIS subtypes, which will be useful for the understanding of the roles of peripheral immune response in different AIS subtypes.

Studies have shown that innate immune cells were initially activated, followed by T cells activation after AIS (37). In addition, the relative levels of CD45RA^+^ and CD45RO^+^ T cells can reveal the systematic immune response and are associated with the pathophysiology and prognosis of multiple disease, such as pancreatic and non-small cell lung cancer (21, 38). In our study, although the lymphocyte count decreased in patients with AIS, the reduced ratio of CD45RA^+^CCR7^+^ (naïve) T cells, and increased ratio of CD45RO^+^CCR7^+^ T (Tcm) and CD45^+^CCR7^−^ T (Tem) cells, further confirmed that the adaptive immune response could be rapidly stimulated in patients with LVO by stimulating the transformation of T cells into memory T cells. Together with the high NLR, the percentage of naïve, Tcm, and Tem can more specifically reflect the immunological condition after AIS with LVO. This is the first study to evaluate naïve, Tcm, and Tem of CD4^+^ and CD8^+^ T cells in peripheral blood of patients with different AIS subtypes.

Considering the critical roles of the immune response, we performed an analysis to quantify and compare the TCR repertoire in PBMCs samples. Analysis of TCR repertoire has been used to characterize various diseases. For instance, the impaired TCR diversity and significant differences in V-J segments in systemic lupus erythematosus (SLE) make the TCR repertoire profile a potential biomarker of SLE (32, 39). Here, we clearly demonstrated that AIS with LVO induced rapid impairment of TCR diversity and the enrichment or reduction of specific V-J combinations in the PBMCs. As the TCR repertoire investigates CDR3, and each CDR3 sequence is a unique label, it can track T cell composition (40). Together with the varied percentage of T cell subsets, the decreased percentage of naïve T cells and segments of TRBV4-1, TRBV5-1, TRBJ1-2, and TRBJ2-2 sequences, as well as the increased percentage of Tcm and Tem cells and segments of TRBV5-3, TRBV6-1, TRBV7-3, TRBV10-1, TRBV13, TRBV23-1, TRBV25-1, TRBJ2-1, and TRBJ2-6 might indicate changes in these sequences in the relevant T cell subsets. In addition, we also found the correlation between amino acid sequence and the severity of AIS with LVO. The combined application of the percentage of Tcm/Tem cells with different abundances of V-J gene combinations and specific amino acid clonotypes could better reflect the body’s immune status in the patients with LVO.

We also found a range of amino acid clonotypes which can be used as a signature for the trained prediction model due to the altered TCR profile. Despite the limited sample size, our model efficiently discriminated AIS patients with LVO from healthy controls, indicating its potential as a biomarker for the diagnosis of AIS with LVO. Currently, the diagnosis of AIS relies mainly on the evaluations of clinical and neuroimaging features, including computed tomography (CT), MRI, and digital DSA (41). However, in the early stage of infarction, mild or no abnormal changes can be found on CT and MRI, because of the low sensitivity of these imaging modalities (42). Although DSA is the gold standard for diagnosing AIS, the expensive cost and invasive operation make its universal application impossible. In addition, all the examinations above require radiation exposure and are not feasible for patients with special circumstances, such as those with a pacemaker or emotional instability. Most importantly, these treatments take a long time and can easily delay the optimal treatment time. Therefore, several studies have been conducted to investigate the rapid diagnostic biomarkers of AIS, including glucose, iron, ferritin, homocysteine, insulin, P-selectin, matrix metalloproteinase-9, high-density lipoprotein cholesterol, platelets, glial fibrillary acidic protein, TNF-*α*, and proenkephalin-A (43–48). However, these biomarkers are not widely used for diagnosing AIS, because of significant individual differences. Moreover, the inflammation-related biomarkers, such as C-reactive protein and interleukin (IL)-6, play a crucial role in predicting AIS (49). Still, they were limited to be used as diagnostic tools because of their similar changes in other inflammatory and infectious processes (50). Taken together, as the rapid changes of TCR repertoire sequences in AIS patients with LVO and the correlation between the CDR3 sequence and LVO severity, our study will provide important assistance for the diagnosis of AIS with LVO. These changes of unique amino acids may be the potential biomarkers for the rapid diagnosis of AIS with LVO.

## Conclusion

In this study, we provided evidence of a change in the peripheral blood cells, the percentage of Tcm/Tem cells, and a predictive role of the TCR repertoire in the AIS with LVO. We found that the LVO group had increased leukocytes, neutrophils and NLR, and decreased lymphocytes as compared to the SVO group, which correlated with the severity of LVO. TCR diversity was impaired in the LVO group, with unique V-J gene combinations, indicating potential biomarkers for LVO diagnosis. Overall, AIS with LVO rapidly triggers a peripheral immune response and our findings will help further understanding of the pathophysiological mechanism of AIS with LVO.

## Data Availability

The datasets presented in this study can be found in online repositories. The names of the repository/repositories and accession number(s) can be found: https://www.ncbi.nlm.nih.gov/, PRJNA1171875.

## Footnotes

^1^ https://www.imgt.org/IMGTrepertoire/LocusGenes/genetable/human/geneNumber.html#TRtotal
