# Supplementary material for: Characteristics of peripheral immune response induced by large-vessel occlusion in patients with acute ischemic stroke
Source: Front Neurol. 2024 Dec 10;15:1512720. doi: 10.3389/fneur.2024.1512720 (PMC11666556; doi:10.3389/fneur.2024.1512720)
Supplement: Supplementary file 6 [file Data_Sheet_1.pdf]

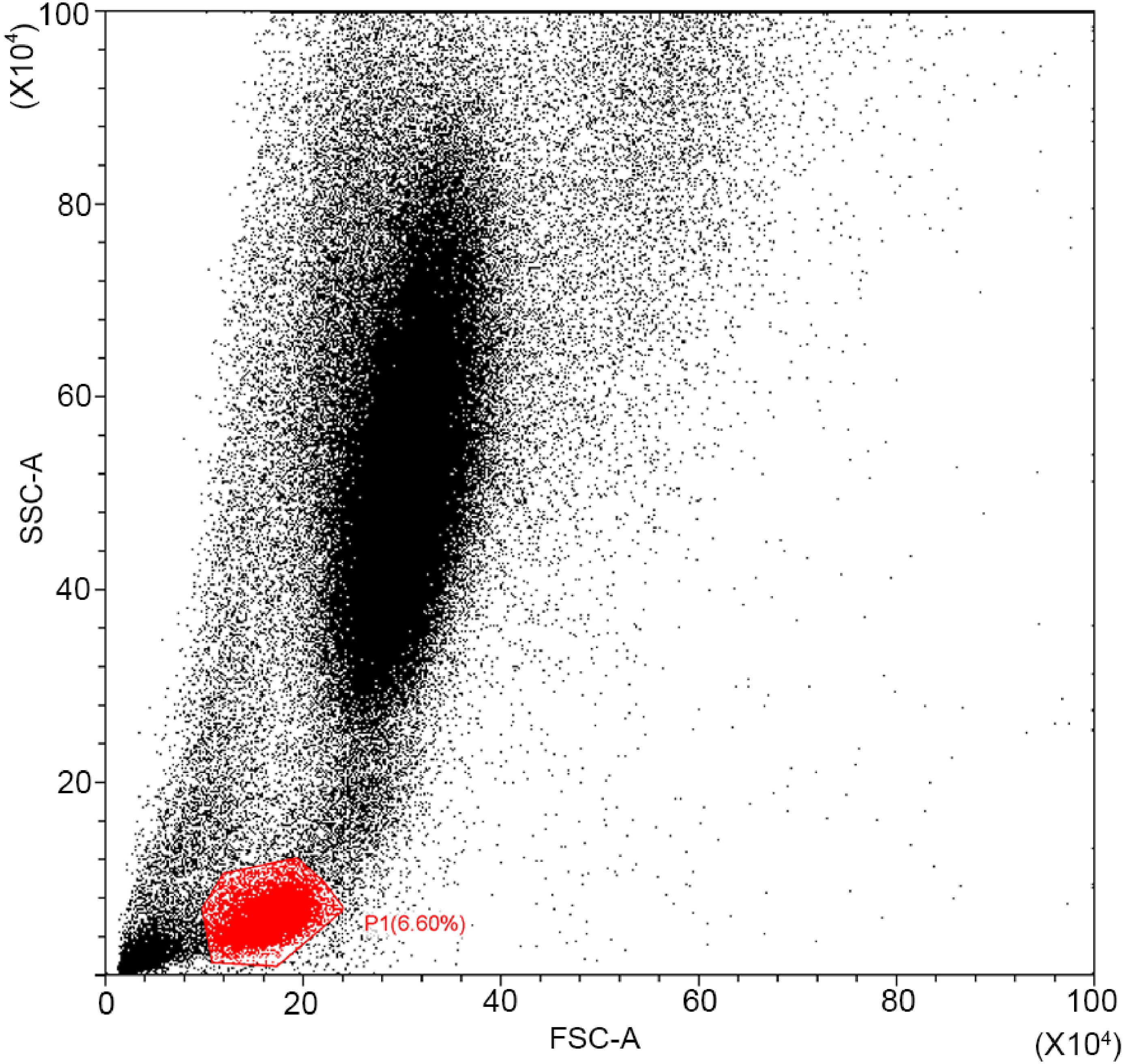

100
(X10⁴)
80
60
SSC-A
40
20
P1(6.60%)
0
20
40
60
80
100
FSC-A
(X10⁴)

Supplemental Figure 1. Gating strategy for the population of lymphocyte in peripheral whole blood. The representative assay was performed with the fresh blood sample of a patient with LVO.
